# Total Trapeziometacarpal Joint Arthroplasty Using the Uncemented HORUS^®^ Prosthesis: Preliminary Results at 6-Month Follow-Up

**DOI:** 10.3390/jfmk11030339

**Published:** 2026-08-28

**Authors:** Rocco Maria Comodo, Silvia Pietramala, Alessio Greco, Riccardo Totti, Giacomo Capece, Giovanni Cepparulo, Rocco De Vitis, Vincenzo De Santis

**Affiliations:** 1 Department of Orthopedics and Geriatric Sciences, Catholic University of the Sacred Heart, Largo Francesco Vito, 8, 00168 Rome, Italy; roccomaria.comodo01@icatt.it (R.M.C.); silvia.pietramala01@gmail.com (S.P.); alessio.greco03@icatt.it (A.G.); riccardo.totti01@icatt.it (R.T.); giovanni.cepparulo01@icatt.it (G.C.); rocco.devitis@policlinicogemelli.it (R.D.V.); 2 Department of Orthopedics, Ageing and Rheumatological Sciences, Fondazione Policlinico Universitario A. Gemelli IRCCS, Largo Agostino Gemelli 8, 00168 Rome, Italy; 3 Department of Orthopaedics, Mater Olbia Hospital, SS 125 Orientale Sarda, 07026 Olbia, Italy

**Keywords:** trapeziometacarpal osteoarthritis, rhizarthrosis, total joint arthroplasty, HORUS prosthesis, thumb carpometacarpal joint, uncemented prosthesis

## Abstract

**Background:** Total trapeziometacarpal (TMC) joint arthroplasty has emerged as a surgical option for selected patients with TMC osteoarthritis. The HORUS^®^ prosthesis (Evolutis, France) is a novel uncemented modular implant featuring a threaded screw-in trapezial cup coated with porous titanium and a PEXEL-E liner (vitamin E-infused cross-linked polyethylene) with a semi-retentive articulation. The purpose of this study was to report preliminary clinical and radiographic outcomes of total TMC joint arthroplasty using the uncemented HORUS^®^ prosthesis at 6-month follow-up. **Methods:** Eleven patients (8 women, 3 men; mean age 63.6 ± 10.1 years) with Eaton-Littler stage II–III TMC osteoarthritis and at least 6 months of unsuccessful conservative treatment underwent total TMC joint arthroplasty using the HORUS^®^ prosthesis. All procedures were performed by a single surgeon through a dorso-radial approach. Clinical outcomes were assessed preoperatively and at 15 days, 1, 3, and 6 months postoperatively using the Visual Analogue Scale (VAS), QuickDASH questionnaire, and Kapandji opposition score. Standardized radiographs were assessed at 1, 3, and 6 months for component position, periprosthetic radiolucency, cup migration/loosening, and stem subsidence. **Results:** One intraoperative trapezial fracture (1/11; 9.1%) required conversion to trapeziectomy with suspension arthroplasty; this patient was excluded from the prosthetic outcome analysis. In the remaining 10 patients, mean VAS decreased from 8.7 ± 1.1 preoperatively to 0.3 ± 0.7 at 6 months, mean QuickDASH decreased from 66.2 ± 11.3 to 14.5 ± 7.6, and mean Kapandji score increased from 5.8 ± 0.8 to 9.2 ± 0.6. One minor postoperative complication occurred (flexor tendinopathy of the third and fourth digits at 4 weeks). No dislocation, infection, or radiographic evidence of component migration, stem subsidence, or periprosthetic radiolucency was detected during the 6-month observation period. **Conclusions:** In this small preliminary series, the HORUS^®^ prosthesis was associated with marked short-term improvements in pain, disability, and thumb opposition. These data should not be interpreted as evidence of long-term implant survival or a reduced risk of loosening because the cohort was small and follow-up was limited to 6 months. Larger comparative studies with longer follow-up are required to assess durability, implant-related complications, and revision risk.

## 1. Introduction

First described by Forestier in 1937, osteoarthritis (OA) of the trapeziometacarpal (TMC) joint is one of the most common sites of degenerative arthritis in the hand after the distal interphalangeal joints [1,2].

It is common in elderly patients and is more prevalent and severe in women than in men [3], with a prevalence of radiographic TMC osteoarthritis reaching approximately 25% in adults aged 55 years and older [4], rising to 33–39% by age 80 [3]. Despite the high prevalence of radiographic changes, only approximately 5–6% of cases become symptomatic [5]. Most patients can be treated conservatively with rest, analgesics, and immobilization [6,7,8], but if conservative management fails, surgery should be considered.

Currently, there are four main types of surgical procedures: trapeziectomy with or without interposition, arthroplasty with interposition implant or resurfacing, trapeziometacarpal fusion and trapeziometacarpal prosthesis (TMP) [9,10,11,12]. To date, no single technique has been conclusively demonstrated to be superior to the others [13,14,15,16,17,18], although a recent network meta-analysis suggests that trapeziectomy with suspensionplasty or ligament reconstruction carries the lowest reoperation risk, while joint replacement achieves the best functional outcomes but with a higher revision rate [19].

The two most widespread alternatives are trapeziectomy and TMP. Trapeziectomy, described in 1949 by Gervis [20], remains the most commonly used procedure due to its efficacy in relieving pain and restoring good thumb range of motion (ROM). However, it is associated with several disadvantages, such as long postoperative recovery time, thumb column shortening, proximal thumb migration, and intracarpal instability [21].

Total trapeziometacarpal joint replacement was first reported by de la Caffinière, with implantations dating back to 1971 [22,23], and has emerged as a surgical alternative over the last two decades. Early implants generally used single-mobility ball-and-socket designs and were associated with substantial complication rates. Subsequent developments have included changes in implant geometry, cementless fixation, and modularity. Recent evidence suggests that total joint arthroplasty may offer short-term functional advantages over trapeziectomy in selected patients. A meta-analysis of randomized controlled trials demonstrated a transient benefit for pain and hand function at 3 months, which diminished to a clinically unimportant difference at 1 year [24,25]. Conversely, a prospective study with a minimum 5-year follow-up reported lower pain and disability scores after total arthroplasty than after trapeziectomy with ligamentoplasty [26,27]. These heterogeneous findings underscore the need to interpret results according to implant design and duration of follow-up.

Several trapeziometacarpal prosthetic systems are currently available, with differences in fixation, cup geometry, bearing design, and mobility. The aim of this study was to describe the preliminary 6-month clinical and radiographic outcomes of the HORUS^®^ prosthesis (Evolutis, France), which, to the best of our knowledge, has not previously been reported in the peer-reviewed literature.

## 2. Patients and Methods

### 2.1. Cohort Characteristics

This study evaluated patients with symptomatic trapeziometacarpal (TMC) osteoarthritis. The study was conducted in accordance with the principles of the Declaration of Helsinki. Ethical review and approval were waived because the analysis relied on pre-existing, fully de-identified and anonymized data. According to institutional and national guidance, research involving completely anonymous datasets that cannot be linked to individual participants does not require formal institutional review board approval. All applicable institutional ethical requirements were followed, and informed consent was obtained from all participants. Patients were eligible if they had Eaton-Littler stage II or III disease [28] and at least 6 months of unsuccessful conservative treatment. Exclusion criteria included occupations or activities associated with high mechanical loading of the thumb, particularly heavy manual work or repetitive forceful pinch activities.

This investigation was a retrospective observational case series based on routinely collected clinical and radiographic data from patients treated during the study period. The analysis was performed after completion of the relevant clinical follow-up using a fully de-identified dataset. Informed consent had been obtained from all participants for the surgical procedure and for the use of their clinical data for research purposes. The retrospective dataset did not contain a specific enrollment log allowing independent verification of consecutive inclusion; therefore, the manuscript does not claim consecutive enrollment.

### 2.2. Preoperative Planning

**Figure 1:**
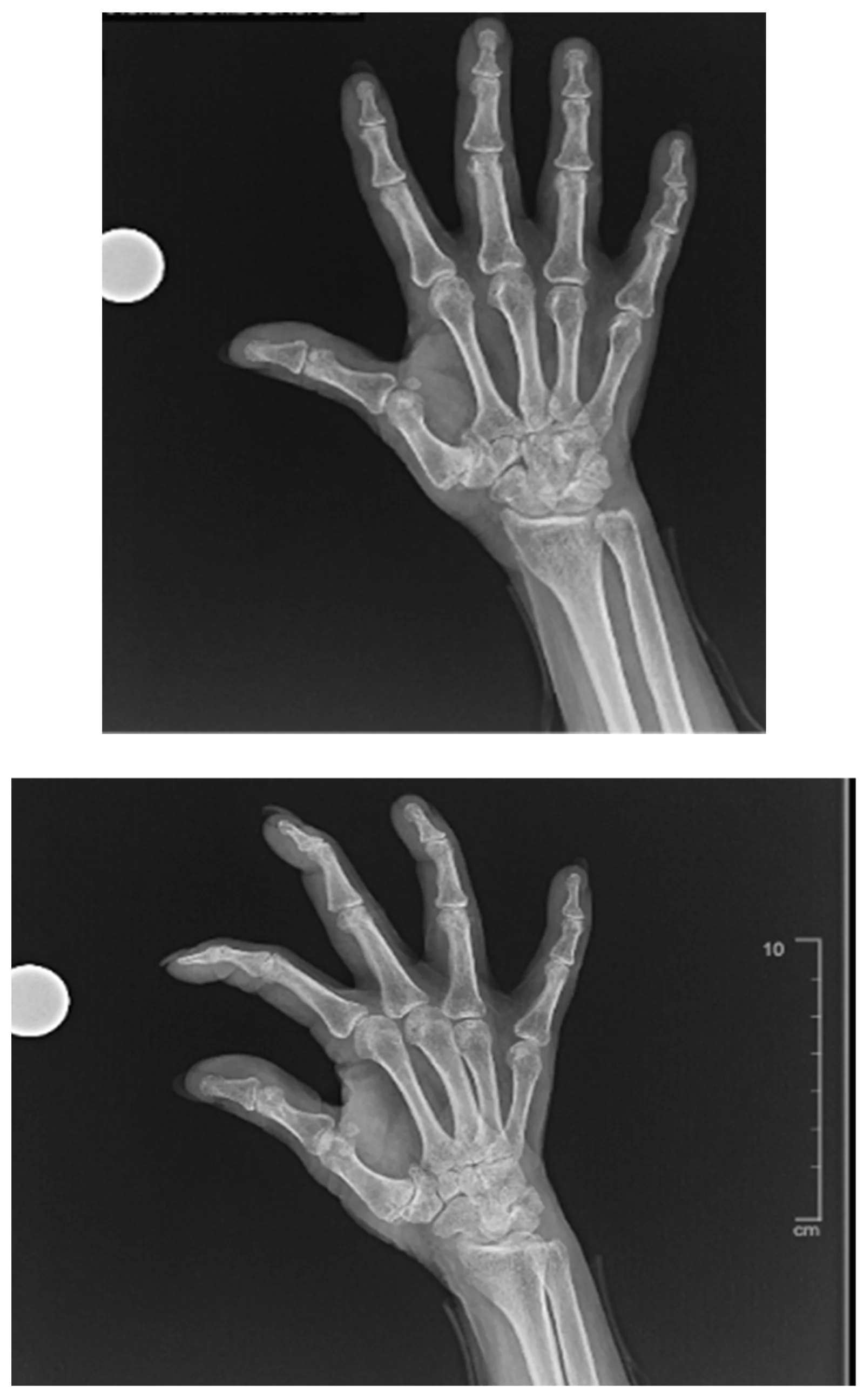
Preoperative radiograph obtained with a 2-Euro coin as a calibration marker.

A standardized preoperative planning protocol was used in all cases. Standard radiographs were obtained with a 2-Euro coin (known diameter, 25.75 mm) as a radiopaque calibration marker for manual measurements on the PACS system. Planning included estimation of trapezial cup size (M or L), metacarpal stem size [1,2,3,4,5], and modular neck length (6 or 8 mm). Trapezial height was also measured to confirm at least 8 mm of bone stock for cup anchorage (Figure 1).

### 2.3. The Implant

**Figure 2:**
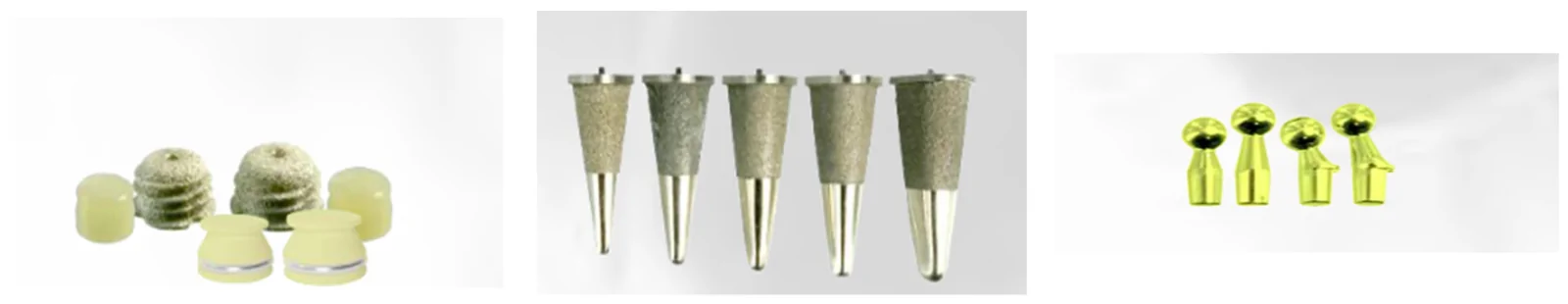
From left to right: cemented and uncemented cups, metacarpal stems (size 1 to 5), standard and 15° offset necks (6 and 8 mm).

All patients received the non-cemented modular HORUS^®^ prosthesis (Evolutis, Briennon, France). The trapezial component features a threaded “screw-in” cup coated with porous titanium (Ti-6Al-4V + porous Ti) to promote secondary biological fixation and maximize primary stability. Although the system offers the option of a cemented trapezial component, a non-cemented threaded cup was preferred and implanted in all cases in this series to exploit the benefits of primary mechanical stability. The system utilizes a PEXEL-E liner (100 mrad cross-linked polyethylene infused with Vitamin E) to minimize wear and optimize sliding properties. The liner includes a built-in mobility chamber designed to reduce pressure on the trapezial cup anchorage. The metacarpal component is an anatomical stem coated with porous titanium for secondary fixation, featuring a proximal anti-migration collar to prevent distal subsidence. The 5 mm diameter femoral-type head articulates with modular Cobalt-Chrome (CoCr) necks, available in straight or 15°-offset configurations (lengths: 6 mm and 8 mm). The modular neck is secured via a Morse taper, providing a resistance nearly twice that of a monoblock neck design. The interface between the neck and the cup is semi-retentive to prevent dislocation, allowing an intra-prosthetic range of motion up to 68° (head-to-neck diameter ratio > 2) (Figure 2).

### 2.4. Surgical Technique

**Figure 3:**
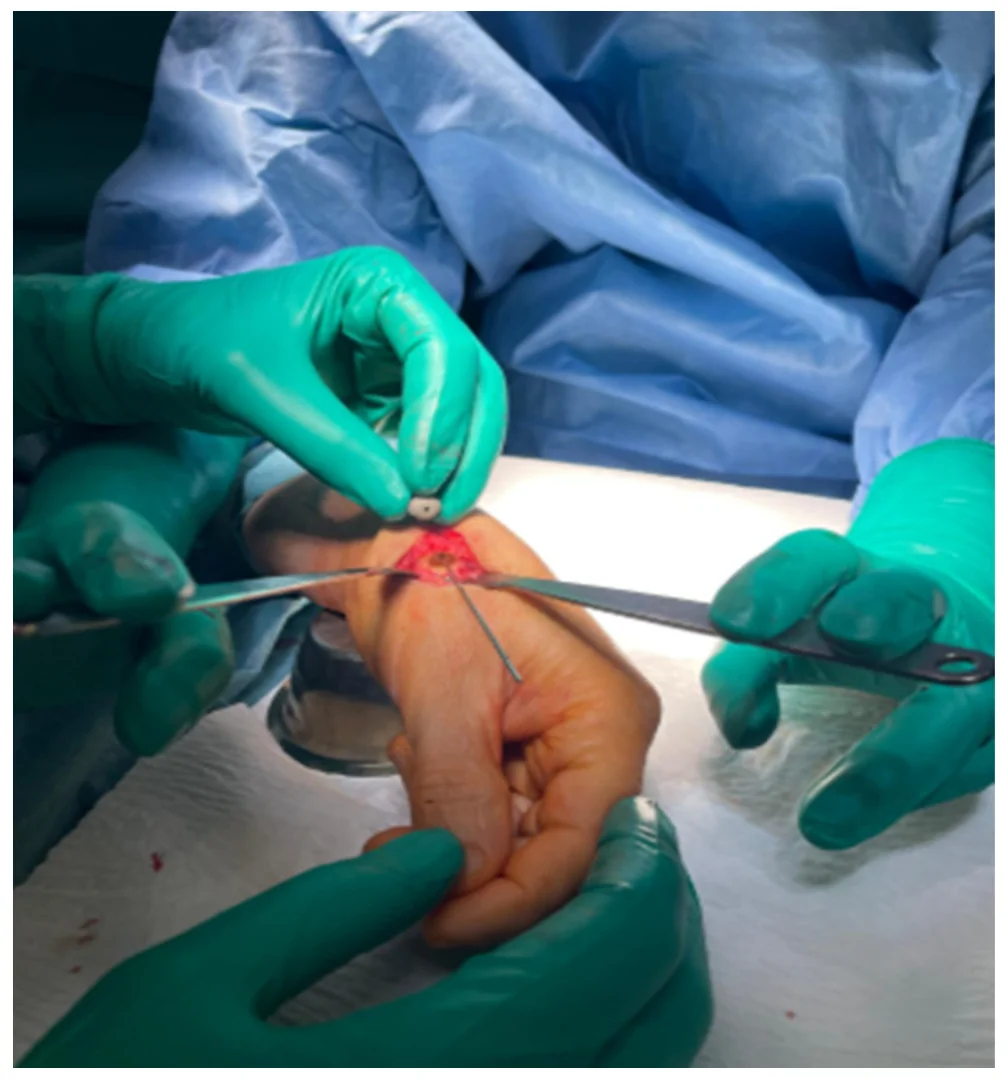
Orientation of the guidewire for cup implantation.

All operations were performed by a single surgeon. All patients received regional anesthesia, and the surgery was performed under tourniquet control. A standard dorso-radial approach was used. During the approach, meticulous dissection was carried out to identify and preserve the sensory branches of the radial nerve and the radial artery, minimizing the risk of postoperative paresthesia or vascular compromise. Careful preservation of the capsule was obtained. A 4 mm resection of the first metacarpal base was performed with an oscillating saw, taking care to remove the ulnar osteophyte when present by an additional resection at a 45° angle. The metacarpal bone was always prepared first, using progressive broaches and leaving the trial stem in situ. Then, the trapezium was prepared using standard technique and orientation (Figure 3). Subsequently, the definitive cup and insert were implanted, and mobility tests were performed with a trial neck. If necessary, an additional bone resection was performed on the metacarpal bone. At this point, the definitive stem was implanted, and new tests were performed with a trial neck. Finally, the definitive neck was implanted. Reduction, irrigation, and suture by layers (capsule and skin) were the last surgical steps. No drains were placed.

Associated procedures were addressed during the same session when needed: carpal tunnel release in 1 case and trigger finger release in 1 case.

### 2.5. Postoperative Management and Rehabilitation

The standardized postoperative protocol involved immobilization in a rigid splint for 2 weeks. At the 2-week mark, sutures were removed, and patients began self-assisted mobilization exercises. Load-bearing was strictly limited to 0.5 kg until the 10th week, followed by progressive loading as tolerated. Patients with painful or adherent scars were treated with a silicone-based scar gel twice daily with deep circular massages.

### 2.6. Clinical and Radiographic Follow-Up

Clinical evaluations were performed at 15 days and at 1, 3, and 6 months postoperatively. Radiographic assessments were performed at 1, 3, and 6 months. All clinical and radiographic assessments were independently reviewed by the same two orthopedic surgeons throughout the study.

Clinical outcomes included pain measured with the Visual Analogue Scale (VAS), upper-limb disability measured with the Quick Disabilities of the Arm, Shoulder and Hand (QuickDASH) questionnaire [29], and thumb opposition measured with the Kapandji score (range, 0–10) [30]. Radiographic assessment was based on standardized postoperative projections, including modified Kapandji views [31]. The immediate postoperative radiograph served as the reference examination. At each subsequent time point, the reviewers qualitatively assessed cup and stem position, the presence or absence of periprosthetic radiolucent lines, interval component migration, and stem subsidence. Radiographic loosening was considered suspected when progressive radiolucency and/or interval component migration was present. Because this was a preliminary series, no radiostereometric or validated quantitative migration analysis was performed; this limitation is acknowledged in the Discussion.

Cup position was assessed by serial comparison with the immediate postoperative radiograph rather than by a prespecified quantitative angular or linear measurement protocol. Accordingly, the analysis was limited to detecting interval change in component position, migration, radiolucency, or subsidence. No objective threshold for defining an optimally positioned or “well-positioned” cup was used, and no claim of optimal cup orientation is made on the basis of these radiographs.

**Figure 4:**
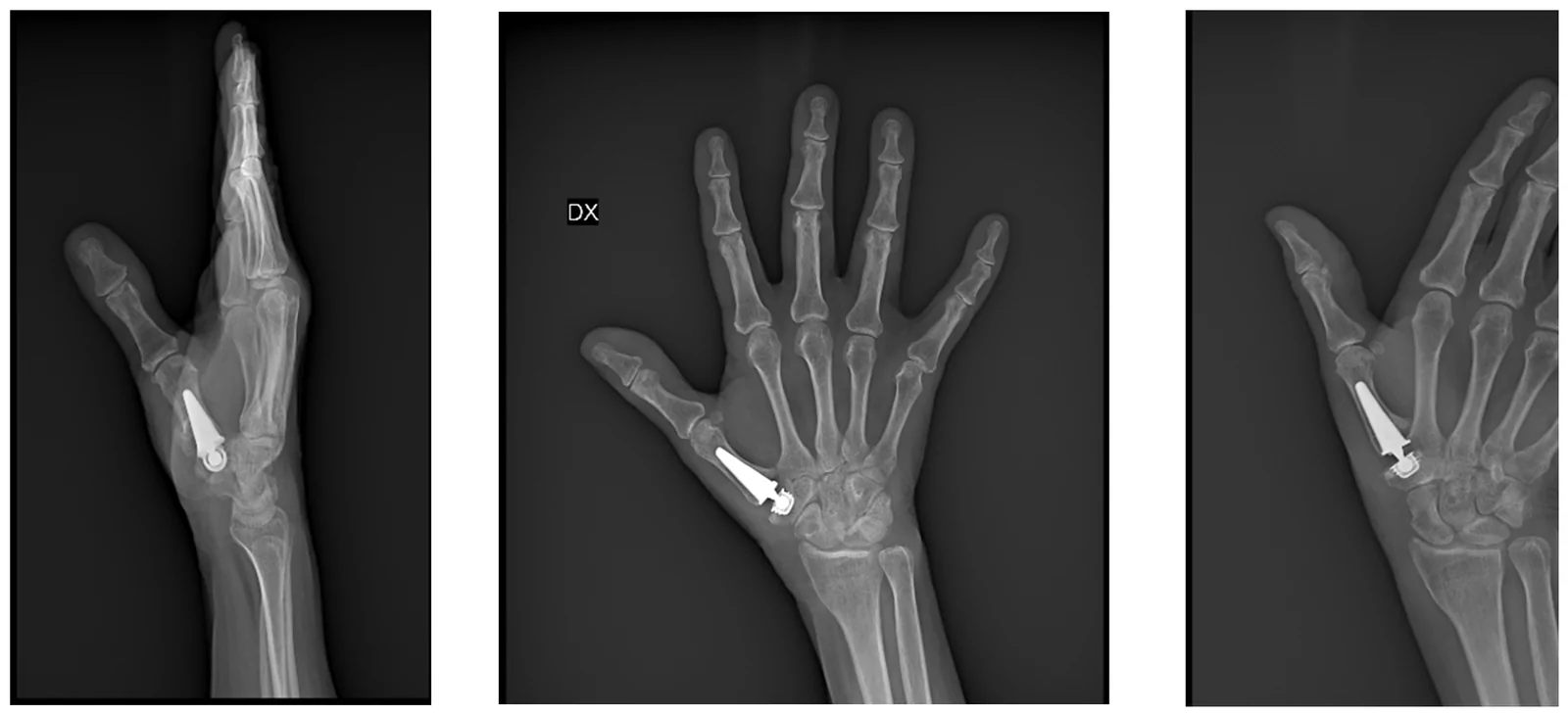
Representative postoperative radiographs showing the HORUS^®^ components in position without evident radiographic complications at the illustrated follow-up.

Thumb opposition was evaluated using the Kapandji score (range, 0–10) [30]. Radiographic outcomes were evaluated at each scheduled imaging follow-up as described above, with particular attention to component position, radiolucent lines, cup migration/loosening, and stem subsidence (Figure 4).

### 2.7. Statistical Analysis

Given the exploratory nature of this preliminary case series and the very small sample size, the statistical analysis was descriptive. Continuous variables are reported as mean ± standard deviation and categorical variables as counts and percentages. No inferential hypothesis testing was performed, and no *p* values were calculated. Changes in VAS, QuickDASH, and Kapandji scores over time are therefore presented as within-cohort descriptive trajectories and should not be interpreted as estimates of treatment effect or comparative efficacy.

## 3. Results

### 3.1. Demographic and Perioperative Data

#### 3.1.1. Baseline Characteristics

A total of 11 patients (8 women, 3 men) underwent surgery for total trapeziometacarpal (TMC) arthroplasty between July 2025 and November 2025. The initial cohort presented a mean age at surgery of 63.6 ± 10.1 years (range: 46–77 years) and a mean body mass index (BMI) of 22.2 ± 1.9 kg/m^2^. Regarding radiological severity, 10 patients were classified as Eaton-Littler stage III, and 1 patient as stage II (Table 1).

#### 3.1.2. Intraoperative Complications and Final Analysis Cohort

One intraoperative complication occurred: a trapezial fracture in a female patient that necessitated immediate conversion to a trapeziectomy and suspension arthroplasty using a modified Weilby technique [32]. This patient followed a dedicated postoperative immobilization protocol (3 weeks of casting followed by 1 week of night splinting) and was excluded from the subsequent prosthetic outcome analysis. Consequently, the final cohort evaluated for prosthetic outcomes consisted of 10 implants in 10 patients (7 women, 3 men), involving 8 right hands and 2 left hands.

For transparency, the intraoperative fracture was retained in the evaluation of procedural feasibility and complications, yielding an intraoperative fracture rate of 1/11 attempted HORUS procedures (9.1%). The patient was excluded only from longitudinal prosthesis-specific outcome calculations because no HORUS implant remained in situ after conversion. Thus, the denominator is 11 for attempted-procedure complications and 10 for postoperative implant-specific VAS, QuickDASH, Kapandji, and radiographic outcomes. This distinction was prespecified in the reporting of the present analysis to avoid treating the conversion as simple loss to follow-up or a successful implantation.

### 3.2. Surgical and Implant Data

For the 10 successful prosthetic implants, the mean operative time was 76.5 ± 11.6 min. Component selection was uniform regarding the cup, with a size M implanted in all cases. Stem sizes varied, with size 2 used in 2 cases, size 3 in 5 cases, and size 4 in 3 cases. A standard neck without offset was used in all implants, measuring 6 mm in 8 cases and 8 mm in the remaining 2 cases (Table 2—Implant Component Selection). Concomitant procedures were performed in two patients: one underwent carpal tunnel release for carpal tunnel syndrome, and another underwent trigger finger release for a fourth-digit trigger finger.

### 3.3. Clinical Course and Pain Control (VAS)

Pain was assessed using the Visual Analogue Scale (VAS). The mean preoperative score was 8.7 ± 1.1. Mean VAS decreased to 4.7 ± 1.5 at 15 days, 3.2 ± 1.5 at 1 month, 0.9 ± 0.9 at 3 months, and 0.3 ± 0.7 at 6 months. At the 6-month follow-up, 8 of 10 patients (80%) reported a VAS score of 0 (Table 3). Given the small sample and descriptive nature of this preliminary series, these values are presented as an observed clinical trajectory rather than evidence of comparative efficacy.

### 3.4. Functional Recovery (QuickDASH)

Upper-limb disability, quantified using the QuickDASH questionnaire, was 66.2 ± 11.3 preoperatively and 65.7 ± 11.9 at 15 days, a time point corresponding to the early immobilization and soft-tissue protection phase. Mean QuickDASH then decreased to 23.0 ± 7.1 at 1 month, 16.9 ± 5.1 at 3 months, and 14.5 ± 7.6 at 6 months (Table 3). These descriptive data are consistent with early functional recovery in this cohort, but no inference regarding superiority over other treatments can be made in the absence of a control group.

### 3.5. Mobility (Kapandji Score)

Thumb opposition was measured using the Kapandji score. The mean score increased from 5.8 ± 0.8 preoperatively to 6.9 ± 0.7 at 15 days, 8.9 ± 0.9 at 1 month, 9.1 ± 0.6 at 3 months, and 9.2 ± 0.6 at 6 months. No patient had clinically evident residual stiffness at the 6-month examination (Table 3).

### 3.6. Radiographic Outcomes

Standardized radiographs, including modified Kapandji views, were reviewed at 1, 3, and 6 months and compared with the immediate postoperative reference images. In all 10 retained implants, component position remained unchanged on serial radiographs, with no visible progressive periprosthetic radiolucent lines, cup migration, or stem subsidence during the 6-month observation period. These findings indicate short-term radiographic stability only; the follow-up duration is insufficient to assess long-term osseointegration, polyethylene wear, or late aseptic loosening.

Because no validated quantitative cup-orientation measurements were obtained, these findings are reported as absence of detectable interval migration or change in position rather than as evidence that the cups met objective criteria for optimal positioning.

### 3.7. Complications

In addition to the intraoperative trapezial fracture described in Section 3.1.2, no prosthetic dislocation, infection, periprosthetic osteolysis, or radiographic evidence of aseptic loosening was observed during the 6-month follow-up. One minor postoperative complication occurred: flexor tendinopathy of the third and fourth digits at 4 weeks, which resolved with conservative management. Because of the small cohort and short follow-up, the absence of additional major complications should not be interpreted as a definitive estimate of implant safety.

## 4. Discussion

Total trapeziometacarpal joint arthroplasty is becoming an increasingly adopted procedure for trapeziometacarpal degenerative arthritis [33], especially in selected patients with high functional demands and those for whom a shorter postoperative recovery is crucial. However, it should be noted that the most recent Cochrane review (2026) concluded that all existing evidence comparing surgical techniques for trapeziometacarpal osteoarthritis remains of “low” to “very low” certainty, and no technique has been definitively proven superior to another [16].

Furthermore, a meta-analysis of randomized controlled trials found that total joint arthroplasty provides only a transient functional benefit over trapeziectomy at 3 months, with differences diminishing to clinically unimportant levels by 1 year [24]. A recent 5-year randomized controlled trial by Bonhof-Jansen et al. (2025) comparing Maïa total joint arthroplasty with trapeziectomy in 62 women found no superiority in patient-reported outcomes on the Michigan Hand Outcomes Questionnaire, although key pinch strength, satisfaction, and willingness to undergo the same treatment again favoured arthroplasty, with 5-year survival of 93% for arthroplasty versus 73% for trapeziectomy [34].

Previous procedures such as suspension arthroplasty led the surgical treatment options for decades; however, despite initial benefits on symptoms and mobility, they showed inferior results at longer follow-up compared to total trapeziometacarpal joint arthroplasty. Simón-Pérez et al. (2025), in a prospective comparative study of 54 patients at a minimum 5-year follow-up, demonstrated that total arthroplasty provided significantly better pain relief (VAS 1.3 ± 0.7 vs. 3.0 ± 0.9; *p* < 0.001) and lower disability scores (DASH 11 ± 9 vs. 28 ± 12; *p* < 0.001) compared to trapeziectomy with ligamentoplasty [26]. Ulrich-Vinther et al. (2008) similarly reported superior early outcomes for prosthetic arthroplasty at 1-year follow-up [35].

The main long-term complication after suspension arthroplasty is progressive proximal migration of the metacarpal base associated with pain. Trapeziectomy procedures are associated with proximal migration of the thumb metacarpal, while joint replacements are associated with implant failure [36]. The first has higher rates in simple trapeziectomy, in which the lack of suspension methods facilitates proximal migration [37]. More recently, the 2026 Cochrane review confirmed that the perceived risk of pain and weakness caused by proximal migration of the first metacarpal, leading to abutment between the thumb metacarpal and scaphoid with loss of thumb ray length, remains a concern with trapeziectomy procedures [16].

The patient sample evaluated in our study involved 8 women and 3 men, with a mean age at surgery of 63.6 ± 10.1 years (range: 46–77 years), reflecting the well-documented female predominance of this pathology and the typical age at which surgical treatment is required. A meta-analysis by van der Oest et al. (2021) confirmed that radiographic thumb base osteoarthritis is more prevalent in females (OR 1.30; 95% CI: 1.05–1.61) and is strongly associated with increasing age (OR 1.06 per year; 95% CI: 1.055–1.065) [3]. Mean age and sex distribution are comparable to those reported in the literature for this surgical procedure [3]. The JAMA review by Currie et al. (2022) further confirmed that thumb carpometacarpal (CMC) joint arthritis is among the most common hand conditions requiring surgical intervention [36]. Rhizarthrosis severity evaluated in our sample with the Eaton-Littler classification showed 10 patients as stage III and 1 patient as stage II; this radiological evaluation, associated with clinical examination, confirms the correct surgical indication to proceed with total trapeziometacarpal joint arthroplasty [8,38].

This study reports only the early 6-month experience with the HORUS^®^ prosthesis. The implant incorporates a threaded uncemented cup, porous titanium coating, a semi-retentive articulation, and a vitamin E-infused cross-linked polyethylene liner. These characteristics provide a theoretical rationale for primary fixation and bearing performance, but the present study was not designed to demonstrate that these design features reduce loosening, wear, or revision risk. The HORUS^®^ prosthesis is manufactured by Evolutis (France), which also produces the ISIS^®^ prosthesis. Fauquette et al. (2023) reported a 94% survival rate in 77 ISIS^®^ implants at a mean follow-up of 107.5 months and found more loosening with cemented than with screwed cups (*p* = 0.0342) [39]. These longer-term ISIS^®^ data provide contextual support for screw-in fixation but cannot be directly extrapolated to HORUS^®^.

Cup loosening is a clinically important complication of total trapeziometacarpal joint arthroplasty because revision may be technically demanding when trapezial bone stock is compromised [40,41,42]. In the present series, no radiographic signs of loosening were identified by 6 months, but this interval is too short to establish the long-term fixation performance of the implant.

Failure of cup fixation may result from inadequate primary stability, cup malposition, unfavourable bone quality, or later loss of secondary fixation related to infection or wear debris. Accordingly, implant geometry is only one of several determinants of stability, and surgical technique and patient anatomy remain important confounding factors.

The HORUS^®^ trapezial component is a porous titanium-coated screw-in cup. Threaded fixation is intended to provide immediate mechanical engagement with the trapezial bone, while the porous coating is intended to support secondary biological fixation. Evidence from other trapeziometacarpal systems suggests that uncemented screw cups can achieve reliable fixation: Hansen and Stilling reported comparable early fixation between cemented and uncemented cups at 2 years [43], whereas Fauquette et al. found less loosening with screwed than with cemented ISIS^®^ cups at longer follow-up [39]. Nevertheless, these results concern other implant systems. The present 6-month HORUS^®^ series cannot determine whether its screw-in geometry confers a lower loosening rate than hemispherical, cemented, press-fit, or other threaded cup designs.

The discussion of threaded fixation, porous coating, surface geometry, and bearing characteristics is therefore intended only to describe the engineering rationale of the device and to place it in the context of existing implant concepts. These theoretical considerations were not directly tested in the present study and should not be interpreted as demonstrated biomechanical or clinical advantages of HORUS^®^.

Nevertheless, a multicenter retrospective study by Farkash et al. [44] valuated complications in 381 patients who underwent total trapeziometacarpal joint arthroplasty and found that inability to achieve primary stability of the cup in the trapezium was the leading cause of failure. However, the great heterogeneity in cup designs, brands, and surgeon expertise are limitations of this study [44].

A point-by-point comparison with established prostheses highlights both the encouraging aspects and the limitations of the present findings. For long-term implant survival, ARPE^®^ has been reported to have approximately 93% survival at 10 years [45,46], whereas MAÏA^®^ series have reported survival of approximately 88% at 10–12 years, with cup loosening and dislocation among the principal causes of revision [47,48]. The ISIS^®^ series reported 94% survival at a mean of nearly 9 years [39]. Dual-mobility Moovis^®^ has shown higher cumulative survival than the single-mobility Elektra^®^ in one comparative series (95.2% vs. 68.6%), with loosening reported in the single-mobility group [47]. Touch^®^ and MAÏA^®^ series also demonstrate substantial improvements in pain and QuickDASH at 1–10 years [47]. In comparison, the HORUS^®^ cohort showed VAS 0.3 ± 0.7, QuickDASH 14.5 ± 7.6, and Kapandji 9.2 ± 0.6 at 6 months, with no radiographic loosening observed over this short interval. Because follow-up durations, patient selection, implant designs, and outcome definitions differ substantially across studies, these values should be viewed as descriptive contextual comparisons rather than evidence that HORUS^®^ performs better than another prosthesis.

The HORUS^®^ bearing uses a PEXEL-E liner consisting of highly cross-linked polyethylene infused with vitamin E. No clinical evidence is currently available for this specific liner in trapeziometacarpal arthroplasty. Vitamin E-infused polyethylene has demonstrated favourable wear behaviour in hip arthroplasty [49,50,51], but the loading environment, component dimensions, and failure mechanisms of the thumb TMC joint differ substantially from those of the hip. Therefore, any potential benefit for HORUS^®^ remains theoretical and requires implant-specific long-term evaluation.

With respect to articulation, the HORUS^®^ system uses a semi-retentive interface. Semi-retentive and non-retentive MAÏA^®^ designs have previously been compared, with lower dislocation risk reported for semi-retentive configurations [52,53]. The HORUS^®^ design allows an intraprosthetic range of motion of up to 68°, but the present cohort is too small to determine whether this feature translates into a lower clinical dislocation rate.

Dual-mobility systems represent an alternative strategy for reducing instability by allowing motion at an additional polyethylene interface. Expert reviews and comparative studies suggest lower dislocation rates with contemporary dual-mobility designs than with older single-mobility implants [54,55,56]. In the comparison by Frey et al., cumulative survival was 68.6% for Elektra^®^ and 95.2% for Moovis^®^, with cup loosening occurring only in the single-mobility group [56]. HORUS^®^ is not a dual-mobility implant; therefore, direct assumptions regarding equivalence or superiority in stability are not justified from our data.

Despite cup design, meticulous positioning of the cup remains a central issue to avoid mobilization: preservation of hard subchondral bone, central cup placement, and cup positioning parallel to the proximal metacarpal surface are crucial to prevent loosening [57,58,59,60].

A size-M cup was used in all 10 retained implants. This uniformity reflects the anatomy of this small cohort and should not be interpreted as evidence that the same cup size is broadly applicable to other populations. Metacarpal stem and neck length were selected according to individual anatomy and intraoperative soft-tissue tension.

The mean operative time for the 10 successful implants was 76.5 ± 11.6 min. Herren et al. (2025), in a learning-curve analysis of 338 Touch^®^ prostheses performed by three surgeons, reported mean operative times of 39, 43, and 59 min, with proficiency achieved after approximately 33–40 cases [61]. The longer operative time observed in our series may reflect early experience with a new implant and instrumentation. However, this explanation remains speculative and should be confirmed in a larger sequential cohort.

One intraoperative trapezial fracture occurred during preparation for cup positioning (1/11; 9.1%). Immediate conversion to trapeziectomy and suspension arthroplasty using a modified Weilby technique was performed, consistent with described salvage strategies [54]. Comparative MAÏA^®^ literature also includes the study by Windhofer et al. [62]. Published fracture rates are lower in larger series: Dass et al. reported 2 fractures among 106 MAÏA^®^ implants (1.9%) [63], whereas Dremstrup et al. reported 9 fractures among 200 Moovis^®^ arthroplasties (4.5%) [54]. The 9.1% rate in our series therefore appears high, although a single event in a cohort of 11 patients produces an unstable percentage estimate. Potential contributing factors described for intraoperative trapezial fracture include limited trapezial bone stock, reduced bone quality, excessive or eccentric reaming, cup oversizing, non-central guidewire/cup positioning, excessive insertion force or torque, and the surgeon’s learning curve [54,57,58,59,60,61]. In the present case, the available retrospective data do not allow one specific causal factor to be established. This event emphasizes the importance of careful preoperative assessment of trapezial dimensions, preservation of subchondral bone, central preparation, and cautious cup insertion. Larger series are required to determine whether fracture risk is related to the HORUS^®^ design, patient anatomy, or the early learning phase.

One postoperative complication was recorded during the 6-month follow-up: flexor tendinopathy of the third and fourth digits, which developed at 4 weeks after surgery. Tenosynovitis is the most frequent minor complication in total trapeziometacarpal joint arthroplasty, occurring in up to 21% of patients [64,65,66]. Herren et al. (2023), in a 2-year follow-up of 101 Touch^®^ patients, reported De Quervain stenosing tenosynovitis (n = 6) and trigger thumb (n = 5) as the most frequent complications [67]. Frey et al. (2025) reported 5 cases of secondary De Quervain tenosynovitis requiring surgery among 88 Touch^®^ prostheses [68]. In particular, De Quervain tendinopathy has been associated with total trapeziometacarpal joint arthroplasty, with a higher frequency in patients undergoing arthroplasty compared to trapeziectomy [69]. According to Ledoux et al., this finding appears related to a preoperative tendinopathy underestimation in patients affected by trapeziometacarpal osteoarthritis. Secondary Linburg-Comstock syndrome post-trapeziectomy surgery involving the thumb and index finger has been reported; however, this secondary tendinopathy has been correlated with trapeziectomy rather than total trapeziometacarpal joint arthroplasty [70].

VAS decreased from 8.7 ± 1.1 preoperatively to 0.3 ± 0.7 at 6 months. For context, Mosegaard et al. reported VAS at rest decreasing from 3.5 to 0.6 and VAS during activity from 7.9 to 2.5 at 12 months [71]; Frittella et al. reported mean VAS values of 2.9 at 3 months and 1.5 at 6 months in 150 dual-mobility prostheses [72]; long-term IVORY^®^ data have shown sustained low pain scores [73]; and Froschauer et al. reported improvement from 8 to 1 at 1 year with Touch^®^ [74]. Long-term comparative outcomes for MAÏA^®^ arthroplasty versus trapeziectomy have also been reported at 9 years [75]. Although the 6-month pain score in our cohort is numerically low, differences in baseline pain, follow-up duration, study design, and implant type preclude conclusions of comparative superiority.

The magnitude of the observed changes in patient-reported outcomes is clinically encouraging, but clinical improvement must be distinguished from statistical inference. No inferential testing was undertaken in this small uncontrolled series, and the study was not designed to establish causality. Consequently, the postoperative improvements cannot be attributed specifically to the HORUS^®^ implant rather than to the surgical procedure, rehabilitation, natural recovery, patient selection, or other unmeasured factors.

QuickDASH improved from 66.2 ± 11.3 preoperatively to 14.5 ± 7.6 at 6 months. Published postoperative QuickDASH values include 19.6 at 10 years with MAÏA^®^ [47], 26.7 at 5 years in another MAÏA^®^ cohort [53], 6.82 at a mean of 1.33 years with Touch^®^ [76], and 14 at a mean of 25 months with Touch^®^ [77]. These data place the observed early functional recovery within the range reported for contemporary implants, but the markedly different follow-up intervals prevent direct efficacy comparisons.

The mean Kapandji opposition score increased to 9.2 ± 0.6 at 6 months. This is similar numerically to values reported with MAÏA^®^ at 5–10 years (8.9–9.2) [47,53] and with dual-mobility prostheses in shorter-term series [72]. This finding supports satisfactory short-term thumb opposition in the present cohort, but it does not establish long-term construct stability or superiority over other implants.

Table 4 provides a descriptive comparison of pain, disability, and opposition outcomes reported for different trapeziometacarpal implants. Because the cited studies differ in follow-up duration, design, sample size, and outcome reporting, the table should be interpreted as clinical context rather than as a formal comparative effectiveness analysis.

### Limitations of the Study

Several limitations must be acknowledged. First, this is a preliminary single-centre series with only 11 operated patients and 10 prostheses available for outcome analysis; consequently, complication percentages are highly unstable and uncommon adverse events cannot be reliably estimated. Second, follow-up was limited to 6 months. This period is sufficient to describe early recovery and detect some early technical failures, but it is inadequate for evaluating long-term osseointegration, polyethylene wear, late migration, aseptic loosening, revision risk, or implant survival. The absence of radiographic loosening at 6 months therefore cannot be extrapolated to longer-term durability. Third, the radiographic assessment was based on serial conventional radiographs and qualitative comparison with the immediate postoperative examination; no radiostereometric analysis or validated quantitative migration protocol was used. Fourth, the single-surgeon design limits generalizability and introduces a potential learning-curve effect. Herren et al. reported that proficiency in TMC arthroplasty may require approximately 33–40 cases [61]. Fifth, the absence of a concurrent control group or direct comparison with an established TMC prosthesis prevents conclusions regarding relative efficacy or safety. Finally, the intraoperative fracture rate was 9.1% (1/11), higher than rates reported in larger series, but the small denominator prevents a stable estimate of true device- or technique-related risk. Future prospective multicenter studies should include larger cohorts, standardized radiographic measurements, a comparator group, and follow-up of at least 5–10 years.

In addition, the retrospective design and the absence of a verifiable consecutive-enrollment log introduce potential selection bias. The analysis was descriptive and was not powered for inferential testing; therefore, neither statistical significance nor implant-specific treatment effects can be inferred from the observed score changes.

## 5. Conclusions

In this preliminary single-surgeon series, total trapeziometacarpal arthroplasty with the uncemented HORUS^®^ prosthesis was associated with marked improvement in pain, QuickDASH, and Kapandji scores over the first 6 postoperative months. Serial conventional radiographs showed no visible component migration, stem subsidence, or progressive periprosthetic radiolucency during this short observation period. One intraoperative trapezial fracture occurred among 11 procedures, yielding a numerically high fracture rate that must be interpreted cautiously because of the small denominator. The present data support the feasibility of the procedure and describe its early clinical course, but they do not establish long-term implant survival, superiority over other TMC prostheses, or a lower risk of loosening. Longer-term, adequately powered comparative studies with standardized quantitative radiographic assessment are required before implant-specific conclusions regarding durability, wear, and revision risk can be made.

## Figures and Tables

**Table 1:** Demographic and Baseline Characteristics.

Patients (n)	11 (10 analyzed)
Sex (F/M)	8/3 (7/3 analyzed)
Mean age ± SD (years)	63.6 ± 10.1
Range (years)	46–77
Mean BMI ± SD (kg/m^2^)	22.2 ± 1.9
Dell/Eaton–Littler Stage II	1
Dell/Eaton–Littler Stage III	10
Mean preoperative VAS ± SD	8.7 ± 1.1
Mean preoperative QuickDASH ± SD	66.2 ± 11.3
Mean preoperative Kapandji ± SD	5.8 ± 0.8

**Table 2:** Implant Component Selection.

Component	Option	n (%)
Cup size	M	10 (100%)
	L	0 (0%)
Stem size	1	0 (0%)
	2	2 (20%)
	3	5 (50%)
	4	3 (30%)
	5	0 (0%)
Neck length	6 mm	8 (80%)
	8 mm	2 (20%)
Neck offset	0°	10 (100%)
Component	15°	0 (0%)

**Table 3:** Clinical Outcomes Over Time.

Outcome	Preoperative	15 Days	1 Month	3 Months	6 Months
VAS (mean ± SD)	8.7 ± 1.1	4.7 ± 1.5	3.2 ± 1.5	0.9 ± 0.9	0.3 ± 0.7
QuickDASH (mean ± SD)	66.2 ± 11.3	65.7 ± 11.9	23.0 ± 7.1	16.9 ± 5.1	14.5 ± 7.6
Kapandji score (mean ± SD)	5.8 ± 0.8	6.9 ± 0.7	8.9 ± 0.9	9.1 ± 0.6	9.2 ± 0.6

**Table 4:** Comparison with the literature.

Study	Prosthesis	N Patients (Prostheses)	Follow-Up (Months)	VAS (Pre → Post)	QuickDASH (Pre → Post)	Kapandji (Pre → Post)
Present study	HORUS^®^	10 (10)	6	8.7 → 0.3	66.2 → 14.5	5.8 → 9.2
Toffoli (2024) [47]	MAÏA^®^	76 (92)	134	–	61.3 → 19.6	– → 9.2
Toffoli (2017) [52]	MAÏA^®^	80 (96)	76	–	61.3 → 17.5	– → 9.2
Andrzejewski (2019) [53]	MAÏA^®^	93 (113)	60	–	– → 26.7	–
Dass (2026) [63]	MAÏA^®^ dual mobility	106	53	7 → 1	–	–
Simón-Pérez (2025) [26]	Touch^®^	26	78	– → 1.3	– → 11	– → 9.9
Lussiez (2021) [66]	Touch^®^	107	≥36	7.4 → 0.8	38 → 20	8.0 → 9.4
Froschauer (2021) [74]	Touch^®^	37 (40)	12	8 → 1	54 → 12	–
Falkner (2023) [77]	Touch^®^	52 (55)	25	–	– → 14	–
Herren (2023) [67]	Touch^®^	130	24	5 → 0	–	–
Tchurukdichian (2020) [73]	IVORY^®^	95 (110)	≥120	– → 0.24	–	–

## Data Availability

All the data we analyzed and tables we compiled are available for any clarification.
